# What Is So Special about Wingsuit BASE Jumpers? A Comparative Study of Their Psychological Characteristics

**DOI:** 10.3390/ijerph19053061

**Published:** 2022-03-05

**Authors:** Pierre Bouchat, Francesco Feletti, Erik Monasterio, Eric Brymer

**Affiliations:** 1 Équipe PErSEUs (EA 7312), Department of Psychology, Université de Lorraine, 57045 Metz, France; 2 Psychological Sciences Research Institute, UCLouvain, 1348 Louvain-la-Neuve, Belgium; 3 Department of Diagnostic Imaging, Ausl Romagna, S. Maria delle Croci Hospital, 48121 Ravenna, Italy; francesco.feletti@polimi.it; 4 Department of Translational Medicine and for Romagna, Università degli Studi di Ferrara, 44121 Ferrara, Italy; 5 Department of Psychological Medicine, Christchurch School of Medicine, University of Otago, Dunedin 8011, New Zealand; erik.monasterio@cdhb.health.nz; 6 Faculty of Health, Southern Cross University, Gold Coast Campus, Lismore 2480, Australia; eric.brymer@scu.edu.au

**Keywords:** extreme sports, personality, sports mental training, sports mental toughness, TCI, wingsuit

## Abstract

For the general public, BASE jumping is considered the ultimate extreme activity. Among BASE jumpers, those using wingsuits are generally perceived as the most experienced but also as the most risk-taking. Starting from this observation, we wanted to know whether wingsuit users differed in their psychological characteristics from other BASE jumpers. More specifically, we hypothesized that wingsuit users would be characterized by higher levels of mental toughness and by lower levels of harm avoidance. We also expected them to use more mental training techniques than the other jumpers. To this end, we conducted a vast survey on a sample of 183 BASE jumpers. Contrary to our hypotheses, the results did not reveal any significant difference in psychological characteristics between wingsuit users and other BASE jumpers. This absence of significant differences is discussed and recommendations for the use of mixed or multi-methods in the study of extreme sports are proposed.

## 1. Introduction

In the last few years, participation in extreme sports has developed rapidly. Perhaps, as a result, research on extreme sports has also experienced exponential increases [1,2]. Different disciplines such as medicine [3,4], psychology [5], sociology [6] and engineering [7] try to understand the determinants of the engagement in these activities, the experience lived by the extreme sports enthusiasts and the effects of these practices at the physiological, psychological, sociological and mechanical levels. This burgeoning body of research on a relatively new theme is exciting but raises a series of fundamental questions for scientists who are playing the role of pioneers. One of them concerns the identification of different categories of participants within the same discipline.

The starting point of this study lies in the observation of different kinds of BASE jump practices (BASE jumping involves parachuting from a fixed point, as summarized by the BASE acronym, which includes the initials of the objects from which jumps are made: buildings, antennas, spans and earth, where spans mean bridges, domes, or arches, whereas earth refers to natural formations, usually cliffs). Despite an increase in the number of participants over the years, BASE jumping remains a niche activity, with around 3000 participants in 2017 based on data from equipment manufacturers [3]. The two main types of BASE jump are jumps from low objects such as antennas, bridges, and buildings (it is conventionally understood that “low jumps” are made from objects of less than 200 m) and jumps from higher objects such as cliffs. Within this second type of jump, one can distinguish different practices, mainly related to the type of outfit used for jumping. There are four main categories of BASE jump “outfits” which are linked to specific kinds of BASE jump practices: 1. Normal clothes (e.g., pants and jacket) are mainly worn by beginners; 2. tracksuits (i.e., pants and jacket designed for inflation) allow a modicum of stability and tracking capacity; 3. onesies (i.e., one-piece tracksuits which increase the tracking capacity and speed); and 4. wingsuits, which transform part of the vertical speed into horizontal speed. The development of high flight glide ratio wingsuits has allowed the introduction of new styles of BASE jumping. On the one hand, the use of wingsuits makes objects jumpable that otherwise would not be. On the other hand, it allows flying down mountain slopes of various conformations, along ridges or through canyons, posing the premise for a new style of flight called proximity flying [8]. Effective tracksuit, onesie and wingsuit piloting is a highly skilled activity [9,10]. 

From an external perspective, a fundamental difference seems to exist between wingsuit users and other BASE jumpers. Indeed, there is a widespread perception that wingsuit BASE jumping is associated with higher risk-taking than other types of cliff jumps. This perception is partly supported by videos of proximity flying, where wingsuit users fly close to objects (e.g., ground, trees, and ridges), up to 250 km/h [11]. A study of BASE jump fatalities between 2007 and 2017 also shows that 61% of fatalities are related to wingsuit BASE jumping, even though wingsuit flying is a relatively minor practice in the discipline [9]. Based on these observations, wingsuit users can be considered as taking more risks than other BASE jumpers, and, also, as much more experienced [8]. Another widely held idea is that the level of expertise required to effectively handle a wingsuit is much higher than other types of flying/falling outfits. All of this contributes to the perception that there are significantly different categories of BASE jump practice associated with different levels of risk-taking. Starting from these insights, we wanted to test whether wingsuit users have different psychological characteristics from other BASE jumpers. Specifically, we focused on psychological factors classically associated with sports practice and risk-taking, such as mental toughness, the existence of specific personality traits and the adoption of psychological strategies, including mental training.

Mental toughness is a personality trait that might be developed following critical incidents, the influence of significant others and targeted interventions [12,13,14,15]. Key attributes of this construct include “coping effectively with pressure and adversity, recovering or rebounding from setbacks and failures, persisting or refusing to quit, being insensitive or resilient, having unshakeable self-belief in controlling ones’ destiny, thriving on pressure and possession of superior mental skills” [16] (p. 165). A vast body of research shows that sport experience and competency in sport are positively related to mental toughness [17,18]. More specifically, elite athletes were found to be more mentally tough than non-elite and amateur ones [19,20,21]. Moreover, evidence suggests that overall mental toughness and specific sub-dimensions of the construct are significantly and positively related to attitudes to risk [16,22]. If we subscribe to the assumptions that wingsuit users are more experienced than the rest of the BASE jumpers and that they take more risks over an extended period of BASE jump practice, we can expect that wingsuit users are characterized by higher levels of mental toughness than other BASE jumpers.

In addition to mental toughness, we wondered whether wingsuit users might possess personality traits that differ significantly from other BASE jumpers. Research on personality traits and participation in high-risk sports is abundant. A recent meta-analysis carried out on 149 effect sizes from 39 articles shows that, compared with either low-risk sport participants or individuals not engaged in any sport, high-risk sport participants were characterized by higher levels of sensation seeking, extraversion and impulsivity [23]. Many recent studies on the personality of extreme sports athletes have been conducted using the Temperament and Character Inventory (TCI) [24,25,26,27,28]. The TCI provides a comprehensive account of personality traits by measuring 7 dimensions of personality [29]: novelty seeking (i.e., tendency to seek out new experiences and active avoidance of frustration), harm avoidance (i.e., propensity to inhibit behaviors and worry about future potential problems), reward dependence (i.e., tendency to maintain behaviors linked to the approval of others), persistence (i.e., tendency to persist in the behaviors despite frustration and fatigue), self-directedness (i.e., will-power, self-determination), cooperativeness (i.e., acceptance and identification of other individuals) and self-transcendence (i.e., feelings of something bigger than the self). Current research has shown that, although BASE jumpers are usually high on novelty-seeking, cooperativeness and self-directedness, and low on harm avoidance and reward dependence, there is no typical or narrowly defined profile of extreme athletes [24,25,26,27]. However, the proposition that wingsuit users are more experienced and take more risks than other jumpers leads us to hypothesize that they are lower in harm avoidance than the other BASE jumpers. 

In addition to personality variables, we were interested in a set of psychological strategies implemented in the context of sports practice. These psychological strategies include mental training, a key factor in the athletes’ performance [30]. Mental training consists of a set of skills such as attention, focus and emotion regulation—and techniques ranging from self-talk to mental imagery. The use of mental training contributes to the building of a stronger mindset through the regulation of stress and emotions [31]. A series of research has indicated that athletes high in mental toughness exhibit more effective use of psychological strategies [17] and are characterized by an enhanced ability to prevent unwanted information from interfering with cognition [21,32]. In summary, if we consider that wingsuit users are more experienced and that they take more risks than the other BASE jumpers, we can expect them to be characterized by higher levels of mental toughness and by lower levels of harm avoidance. Based on anecdotal evidence among the BASE community, we could also expect them to use more mental training techniques than the other jumpers. 

Starting from the insights that wingsuit BASE jumping is both riskier and requires more experience than other types of BASE jump practices, we made three main hypotheses regarding the psychological characteristics of wingsuit BASE jumpers compared with non-wingsuit BASE jumpers.

**Hypothesis** **1** **(H1).** We expect wingsuit BASE jumpers to be characterized by higher levels of mental toughness than other BASE jumpers.

**Hypothesis** **2** **(H2).** We hypothesize that wingsuit BASE jumpers should present lower levels of harm avoidance than the other BASE jumpers.

**Hypothesis** **3** **(H3).** We expect wingsuit users to use more mental training techniques than the other jumpers.

## 2. Method

### 2.1. Procedure and Participants

A vast online survey was designed to address our hypotheses. English and French versions of the questionnaire were distributed through Facebook BASE jump groups and to the personal network of the main researcher, who is a BASE jumper himself (Regarding the French version of the questionnaire, validated French versions of most scales were used. For measures where no French validation was available, items were translated and then back-translated by a native-speaking researcher in psychology). A total of 183 BASE jumpers completed the questionnaire between 14 February and 2 March 2020. Besides one observation which was removed due to its unrealistic pattern of answers, no outlier was excluded. To test our hypotheses, participants were divided into two subsamples: Wingsuit BASE jumpers and “other” BASE jumpers. Subsamples’ characteristics are available in Table 1. Formal ethical approval has been obtained from the Faculty Ethics Committee from Université libre de Bruxelles (Comité d’Éthique Facultaire, affiliated with the Faculté des Sciences Psychologiques et de l’Éducation). 

### 2.2. Measures

Completing the questionnaire took, on average, 15 min. Most indicators consisted of short, adapted versions of existing scales to maximize the number of answers. Unless specified, all items were rated on 5-point scales ranging from 1 (“not at all”) to 5 (“very strongly”). In addition to demographics, participants were asked to answer the questions listed in the following paragraphs.

Mental Toughness has been measured using an adapted short version of the Sports Mental Toughness Questionnaire [12,33]. It was composed of 9 items covering the three main dimensions of mental toughness: Confidence (e.g., “I have an unshakeable confidence in my ability”; α = 0.80), Constancy (e.g., “I take responsibility for setting myself challenging targets”; α = 0.74) and Control (e.g., “I get angry and frustrated when things do not go my way”; α = 0.71). A total score of Mental Toughness was also computed (α = 0.57).

Temperament and Character Inventory’s seven dimensions were measured using a short version in 56 items [34,35]. Given their good internal consistency, seven indicators were computed: novelty seeking (α = 0.57), harm avoidance (α = 0.77), reward dependance (α = 0.77), persistence (α = 0.71), self-directedness (α = 0.68), cooperativeness (α = 0.72) and self-transcendence (α = 0.89).

Sports Mental Training was measured using a 10-item adapted version of the Sports Mental Training Questionnaire [31]. Four of the five factors were included: Foundational Skills (e.g., “I know my own value, my strengths, and weaknesses, and I plan how to improve them”; α = 0.67), Performance Skills (e.g., “When I am under pressure, I’m able to relax physically and mentally, so that I am ready to perform”; r = 0.47), Self-Talk (e.g., “I use self-talk to help myself overcome difficult times”; r = 0.56) and Mental Imagery (e.g., “During preparation for the jump I create real and accurate “inner films” planning possible obstacles and feeling sensations associated with the actual situation to come”; α = 0.70). A total score of mental training was also computed (α = 0.77).

## 3. Results

We voluntarily departed from complex multivariate analyses, as the main objective of this paper is descriptive comparative, to highlight the psychological differences between wingsuit users and the other BASE jumpers. As such, the use of comparison analyses seems especially appropriate. In a first step, we focused on demographic variables. Results of chi-squared and Welch *t*-tests show that, although wingsuit users and the other BASE jumpers do not significantly differ in terms of age and gender balance (We decided to test whether gender was a significant discriminant variable within the two jumper samples. Welch’s *t*-test results show that within the two groups of jumpers, women and men differ on only one characteristic: participation in other risky activities. In the wingsuit group, women engage in significantly more other risky activities than men. The pattern is opposite for the other jumpers. Other than that, men and women do not differ on any other variable. Based on these results, we decided to compare both groups (wingsuit users and other jumpers) regardless of their gender in the subsequent analyses), the former carry out practice for longer, jump more often and have significantly more BASE jumps than their counterparts (see Table 1). These results tend to confirm that wingsuit BASE jumpers are more experienced than the other jumpers. Interestingly, they also report fewer risky practices outside BASE jumps than the other jumpers (These self-reported practices are highly varied: Speed flying, paragliding, skydiving, mountaineering, rock climbing, big wave surfing, kite surfing, diverse forms of diving (e.g., cave, apnea) and highlining. Interestingly, practices such as walking alone, cycling in a city and sex are also mentioned by a series of participants. The mention of these last practices suggests a surprisingly realistic perception of risk-taking). 

The values in brackets in the two first columns are Standard Deviations. The values in brackets in the last column are effect sizes.

In a second step, Welch *t*-tests were run to grasp the potential psychological differences between wingsuit BASE jumpers and non-wingsuit BASE jumpers. We used non-parametric tests given the imbalance in the subsamples’ sizes [36]. Contrary to all our hypotheses, the results of the comparison tests show no significant differences but one between wingsuit users and non-wingsuit BASE jumpers. The only exception to this pattern is the level of self-talk which is significantly lower among wingsuit users than among the other jumpers (see Table 2).

In a third step, given the absence of significant differences in personality and mental training dimensions between wingsuit and non-wingsuit BASE jumpers, we investigated from an exploratory point of view whether, instead of differences between categories of jumpers, there would be differences within wingsuit BASE jumpers related to experience. To carry this out, we conducted bivariate correlations to see if wingsuit users’ experience level was related to their psychological characteristics. Results of the correlations show that, besides one dimension of mental toughness and one personality trait (i.e., persistence), there were no significant correlations between the number of wingsuit BASE jumps and our variables of interest (see Table 3).

## 4. Discussion

The purpose of this study on the largest sample of BASE jumpers mobilized to date was to consider potential psychological differences that may exist between wingsuit users and other jumpers. Based on the widespread perception that wingsuit BASE jumping is associated with higher risk-taking than other types of cliff jumps and that wingsuit users would be more experienced than other jumpers, we expected the former to differ from the latter on several psychological factors. More specifically, we hypothesized that wingsuit users would be characterized by higher levels of mental toughness and by lower levels of harm avoidance. We also expected them to use more mental training techniques than the other jumpers. Our results, however, did not reveal the existence of distinct profiles in terms of these psychological variables. This relative lack of empirical evidence of psychological differences between wingsuit users and other BASE jumpers can be explained in at least 3 ways. First, despite actual differences in “objective” experience (i.e., number of years of practice, number of total jumps, frequency of practice), wingsuit users would not actually differ from other jumpers in terms of psychological characteristics. Secondly, it is also possible that many participants who were other jumpers may eventually go on to become wingsuit users with more time in the sport and therefore confound the differences in temperament across both groups. A third explanation is that our research method and measurement tools were not able to capture such differences. We will develop these hypotheses in the remainder of the discussion.

### 4.1. A False Dichotomy?

This first option is that wingsuit users and the other BASE jumpers would not substantially differ in terms of personality and mental characteristics. Another possible interpretation is that psychological variables other than those we measured could explain the differences in practice. For instance, the perception of risk may be associated with the different types of practice [37,38], the emotions felt during the edge experience [39,40] and the feeling of self-efficacy [41] concerning the practice of BASE jumping. Further, the fact that wingsuit users were less involved in participation in other risk-taking activities might suggest that obsessionality is an important variable to take into account in future studies. Nevertheless, what our results suggest is that the distinction between elite (in this case the wingsuit users) and amateur athletes (in this case the other jumpers) seems less relevant in BASE jumping than in non-risk sports. In the latter, high-achievement athletes are frequently distinguished from amateurs in terms of their psychological characteristics [42,43,44,45,46]. In BASE jumping, moving into wingsuit BASE may be a natural progression from extended practice and a result of opportunity, rather than based on participants’ psychological characteristics and that effective performance in wingsuit BASE jumping is likely to require a very similar set of characteristics as effective performance in other BASE disciplines. 

### 4.2. A Plea in Favor of Mixed or Multi-Methods

In the case of this study, the use of standardized questionnaires and basic quantitative analyses (statistical methods such as latent profiles analyses could have been used if sample’s size was close to 500 [47] did not allow us to capture the psychological specificities of different types of BASE jumpers. We have hypothesized above that this lack of observation of differences may be due to a real absence of psychological specificities. However, since the non-observation of statistically significant differences does not mean that there are no real differences, the use of additional data collection methods, statistics (an important limitation of the present study lies in the lack of multigroup measurement invariance analysis due to small sample sizes [48]. Treating the French- and English-speaking subsamples as a whole might have influenced the final results of our analyses), and even, of different psychological models, would have been of considerable value. History has shown on several occasions that while extrapolating ideas from one field to another field can have broad application, it is often the case that important nuances are missed. In sport, a good example is the early extrapolation of knowledge from mainstream psychology to sport psychology [49]. Although research in the early days was broadly helpful, important nuances were missed which triggered the move to the development of sport-specific models. Arijs and colleagues [50] argued that extreme sports need to be recognized as different enough from mainstream sport that specific models need to be developed for extreme sports. The need for different models is often accompanied by the need for different research methods. The present study highlights the limits of purely quantitative designs and suggests that qualitative methodologies able to draw out nuances are important [50]. Challenges for research in this area include the fact that quantitative and qualitative research are often framed by different ontological and epistemological frameworks [51]. Although many have argued that truly mixed methods are potentially incompatible, studies that utilize one or the other or collaborative studies that draw on both might provide the evidence required to produce models suitable for extreme sports. One example of this practice is a study by Monasterio and Brymer [52] which adopts an autoethnographic approach to explicate a rock-climbing accident. Their findings show that beyond personality factors—traditionally measured through quantitative questionnaires—effective climbing is also determined by the reinforcement of humility and self-awareness. 

## 5. Conclusions

The current research addressed the psychological specificities of wingsuit users compared with the other BASE jumpers. Our results showed that wingsuit users and the other jumpers displayed quite similar psychological patterns. We tried to explain these results in at least three ways, one of which constitutes a plea in the favor of the use of mixed methods for studying the extreme sports experience. In our view, the combination of understanding real-life motivators and decision-making of individuals, with quantitative data in populations, is the goal of longer-term research and will enrich not only extreme sports research but also research into complex behaviors.

## Figures and Tables

**Table 1:** Samples’ Characteristics.

Measures	Wingsuit BASE Jumpers *N* = 61	Others *N* = 121	Chi-square and *t*-Tests
Gender	7 women	8 women	1.27 (0.08)
Age	38.41 (8.63)	36.24 (9.87)	−1.52 (0.23)
Years of practice	7.98 (5.75)	4.57 (5.15)	−3.91 *** (0.64)
Frequency of practice (days/year)	70.52 (59.06)	48.79 (51.36)	−2.44 * (0.40)
Number of BASE jumps	532.13 (439.46)	259.80 (367.16)	−4.16 *** (0.69)
Other risky activity than BASE	Yes (75.4%)	Yes (87.6%)	−1.93 ^a^ (0.33)

Note: ^a^ *= p =* 0.057; * = *p* < 0.05; *** = *p* < 0.001.

**Table 2:** Descriptive Statistics and Comparison Tests for the Main Variables Considered.

Measures	WS Base Jumpers *n* = 61	Others *n* = 121	Welch *t*-Test Value	Effect Size (Cohen’s d)
Mental Toughness—Confidence	3.699 (0.759)	3.548 (0.725)	−1.288 (116)	−0.204
Mental Toughness—Constancy	4.022 (0.647)	4.014 (0.664)	−0.079 (123)	−0.012
Mental Toughness—Control	2.738 (0.876)	2.625 (0.815)	−0.836 (113)	−0.133
Mental Toughness—Total	3.661 (0.547)	3.645 (0.502)	−0.187 (112)	−0.030
Mental Training—Foundational Skills	3.811 (0.822)	3.774 (0.722)	−0.300 (105)	−0.048
Mental Training—Performance Skills	4.067 (0.751)	3.933 (0.788)	−1.105 (123)	−0.173
Mental Training—Self-Talk	2.867 (1.116)	3.300 (1.127)	2.448 * (119)	0.386
Mental Training—Mental Imagery	3.722 (0.961)	3.878 (0.792)	1.083 (100)	0.177
Mental Training—Total	3.647 (0.660)	3.743 (0.580)	0.961 (106)	0.155
TCI—Cooperativeness	3.936 (0.603)	4.062 (0.597)	1.330 (119)	0.209
TCI—Self-Transcendence	2.557 (0.969)	2.481 (1.087)	−0.479 (133)	−0.074
TCI—Self-Directedness	3.924 (0.615)	3.996 (0.612)	0.744 (120)	0.117
TCI—Reward- Dependence	3.189 (0.760)	3.351 (0.724)	1.385 (115)	0.219
TCI—Harm Avoidance	2.389 (0.719)	2.413 (0.691)	0.214 (116)	0.034
TCI—Persistence	3.762 (0.622)	3.798 (0.596)	0.365 (116)	0.058
TCI—Novelty-Seeking	3.680 (0.589)	3.766 (0.623)	0.907 (127)	0.141

Note: * *p* = 0.016; Scales ranged from 1 to 5 (see above). With a single exception, mean levels of all variables of interest do not significantly differ between wingsuit users and other jumpers. The values in brackets in the two first columns are Standard Deviations. The values in brackets in the third column are degrees of freedom of Welch *t*-tests.

**Table 3:** Correlations Between the Number of Wingsuit BASE Jumps and Variables of Interest.

Measures	Number of Wingsuit BASE Jumps
Mental Toughness—Confidence	−0.056
Mental Toughness—Constancy	−0.406 **
Mental Toughness—Control	−0.022
Mental Toughness—Total	−0.079
Mental Training—Foundational Skills	−0.198
Mental Training—Performance Skills	0.004
Mental Training—Self-Talk	−0.090
Mental Training—Mental Imagery	−0.203
Mental Training—Total	−0.192
TCI—Cooperativeness	0.003
TCI—Self-Transcendence	0.038
TCI—Self-Directedness	−0.068
TCI—Reward-Dependence	−0.082
TCI—Harm Avoidance	0.069
TCI—Persistence	−0.363 *
TCI—Novelty-Seeking	0.007

Note: Sig. Two-tailed: * *p* < 0.05. ** *p* < 0.01; Correlations between variables have been calculated separately for the WS BASE subsample.

## Data Availability

The dataset, questionnaire, and ethical approval form have been made publicly available on the OSF and can be accessed at https://osf.io/umc7j/?view_only=e40404996f534ce0a0336ae7d5214081 (accessed on 20 January 2022).
